# *Pseudomonas aeruginosa *biofilm dispersion by the mouse antimicrobial peptide CRAMP

**DOI:** 10.1186/s13567-022-01097-y

**Published:** 2022-10-08

**Authors:** Yang Zhang, Peng Cheng, Shiyuan Wang, Xiaofen Li, Lianci Peng, Rendong Fang, Jing Xiong, Hui Li, Cui Mei, Jiye Gao, Zhenhui Song, Dengfeng Xu, Lizhi Fu, Chenghong Li, Xueqing Wu, Yuzhang He, Hongwei Chen

**Affiliations:** 1 College of Veterinary Medicine, Southwest University, Chongqing, 402460 China; 2 Immunology Research Center, Medical Research Institute, Southwest University, Chongqing, 402460 China; 3 National Center of Technology Innovation for Pigs, Chongqing, 402460 China; 4 Chongqing Academy of Animal Sciences, Chongqing, 402460 China; 5 Department of Infectious Diseases, Sir Run Run Shaw Hospital, Zhejiang University School of Medicine, Hangzhou, 310020 China

**Keywords:** *Pseudomonas aeruginosa*, biofilm dispersion, antimicrobial peptide, CRAMP, c-di-GMP, PQS system, omics data

## Abstract

*Pseudomonas aeruginosa* (*P. aeruginosa*) is a known bacterium that produces biofilms and causes severe infection. Furthermore, *P. aeruginosa* biofilms are extremely difficult to eradicate, leading to the development of chronic and antibiotic-resistant infections. Our previous study showed that a cathelicidin-related antimicrobial peptide (CRAMP) inhibits the formation of *P. aeruginosa* biofilms and markedly reduces the biomass of preformed biofilms, while the mechanism of eradicating bacterial biofilms remains elusive. Therefore, in this study, the potential mechanism by which CRAMP eradicates *P. aeruginosa* biofilms was investigated through an integrative analysis of transcriptomic, proteomic, and metabolomic data. The omics data revealed CRAMP functioned against *P. aeruginosa* biofilms by different pathways, including the Pseudomonas quinolone signal (PQS) system, cyclic dimeric guanosine monophosphate (c-di-GMP) signalling pathway, and synthesis pathways of exopolysaccharides and rhamnolipid. Moreover, a total of 2914 differential transcripts, 785 differential proteins, and 280 differential metabolites were identified. A series of phenotypic validation tests demonstrated that CRAMP reduced the c-di-GMP level with a decrease in exopolysaccharides, especially alginate, in *P*. *aeruginosa* PAO1 biofilm cells, improved bacterial flagellar motility, and increased the rhamnolipid content, contributing to the dispersion of biofilms. Our study provides new insight into the development of CRAMP as a potentially effective antibiofilm dispersant.

**Supplementary Information:**

The online version contains supplementary material available at 10.1186/s13567-022-01097-y.

## Introduction

Biofilms have been widely recognized as the dominant mode of bacterial growth in nature [1]. Bacterial biofilms are surrounded by extracellular polymeric substances (EPS) as a diffusive barrier, inhibiting traditional antibiotics from penetrating deeply [2]. The major components of EPS are exopolysaccharides (which are sometimes called EPSs), proteins, extracellular DNA, lipids, and other biopolymers [3]. In addition, EPSs generate nutrient gradients for biofilms, which can reduce the growth rate and metabolic activities of bacterial biofilms, causing an increase in the number of persister cells [3]. It has been extensively reported that the tolerance of biofilms to various antibiotics is 10–1000 times greater that of planktonic cells [1, 2, 4]. The well-known opportunistic pathogen *P. aeruginosa*, which exhibits a strong biofilm formation ability, can cause infection in different species, including humans, animals, and plants [5]; furthermore, *P. aeruginosa* is a main pathogen in cystic fibrosis in human clinical practice. Recently, the number of clinically cases in which animals are infected by *P. aeruginosa* has also increased year by year, and *P. aeruginosa* infection is more prevalent in small animals than in large animals [6]. *P. aeruginosa* can cause haemorrhagic pneumonia in minks, which mainly leads to great economic losses [7]. Furthermore, *P. aeruginosa* is the main culprit of otitis media and pyoderma in dogs and cats, and these diseases are accompanied by serious antibiotic resistance [8, 9]. It is necessary to always observe the changing trend in the antibiotic resistance pattern of *P. aeruginosa* and be alert to cross infection and pathogen spread between pets and their owners [6]. *P. aeruginosa* has been classified as an antimicrobial-resistant ESKAPE (*Enterococcus faecium*, *Staphylococcus aureus*, *Klebsiella pneumoniae, Acinetobacter baumannii*, *Pseudomonas aeruginosa*, and *Enterobacter *spp.) pathogen [10]. In 2017, the World Health Organization listed *P. aeruginosa* as one of the most critical pathogens for which a new antibiotic is urgently needed [2, 3]. Furthermore, *P. aeruginosa* is also a model organism for the study of biofilm formation [3].

The life cycle of biofilms includes the following stages: reversible/irreversible adhesion, formation, maturation, and dispersion [1]. A previous study concentrated on the inhibition of biofilm formation, while the underlying mechanism by which bacteria disperse from the biofilm has rarely been investigated in recent years [1]. Dispersion is generally characterized as the terminal stage of biofilm development [1]. Inducing biofilm dispersion could be a promising strategy to combat biofilm-related infections, as the dispersed cells and remaining biofilm cells are more vulnerable to antimicrobial agents and immune responses [1, 11]. Biofilm dispersion is divided into two forms, including active dispersion and passive dispersion [11, 12]. Active dispersion depends on a reduction in the intracellular cyclic dimeric guanosine monophosphate (c-di-GMP) level, resulting in the production of enzymes that degrade the biofilm matrix and promote dispersion. However, passive dispersion relies on external triggers, leading to the release of single cells or clumps of biofilms [11]. Research on biofilm dispersion will be advantageous for the development of antibiofilm products, thereby contributing to the effective control of infections caused by biofilm formation.

Antimicrobial peptides (AMPs) are potent, highly cationic, and amphiphilic broad-spectrum host defence antimicrobials and are significant for the next generation of antibiotics [2, 13]. AMPs can mainly kill bacteria through physical damage and inhibit the development of antibiotic resistance [2]. Moreover, AMPs have been recently found to present antibiofilm effects by suppressing biofilm formation or eradicating previously formed biofilms [13–18]. However, few studies have concentrated on the mechanism by which AMPs disperse biofilms. We have recently reported that the mouse homologue cathelicidin-related AMP (CRAMP) could inhibit the formation of *P. aeruginosa* PAO1 biofilms at subminimum inhibitory concentrations (MICs) [19] and reduce the biomass of preformed *P. aeruginosa* 27 853 biofilms [20]. More importantly, in our studies, CRAMP was the only AMP that could eradicate the *P. aeruginosa* biofilm, while others could only kill part of the bacterial biofilm [20].

In the present study, we attempted to further confirm the role of CRAMP in eradicating preformed PAO1 biofilms by measuring the biofilm biomass, viable count of biofilms, and visualized biofilms via confocal laser scanning microscopy (CLSM). The results of Kyoto Encyclopaedia of Genes and Genomes (KEGG) pathway analysis revealed that the effect of CRAMP on eradicating biofilms was mainly attributed to a series of biofilm formation-associated phenotypes triggered by the reduced c-di-GMP level. In addition, the relevant phenotypes of dispersed bacteria were also validated, confirming that CRAMP could be used as a potentially effective dispersal agent against biofilms.

## Materials and methods

### Anti-biofilm testing and preparation of biofilm samples

*Pseudomonas aeruginosa* PAO1 was purchased from the China General Microbiological Culture Collection Center (Shanghai, China). Strains were grown overnight at 37 ℃ in Mueller Hinton (MH) broth, centrifuged, diluted to 0.1 optical density at 600 nm (OD_600_) and then used as the test bacterial solution by diluting 100 times. The biofilms were preformed by adding the test bacterial solution into 96-well plates (Corning^®^3599; Corning Inc., Corning, NY, USA) and incubating at 37 ℃ for 24 h. Afterwards, the plates were washed three times with phosphate-buffered saline (PBS). The twofold dilutions ranging from 4 MICs to 1/16 MIC for CRAMP (synthesized by ChinaPeptides Co., Ltd., Shanghai, China; Additional file 1E), LL-37 (synthesized by ChinaPeptides Co., Ltd.; Additional file 1E), ciprofloxacin (CIP; Shanghai Yuanye Bio-Technology Co., Ltd., Shanghai, China), and gentamicin (GEN; Shanghai Yuanye Bio-Technology Co., Ltd.) was prepared with sterile water. Next, 100 µL of each concentration was added to the corresponding plate and incubated at 37 ℃ for 1 h. The appropriate concentration of CRAMP was selected and tested in a 6-well plate (Corning^®^3516; Corning Inc.) and cell culture flasks (25 cm^2^; Corning^®^430168; Corning Inc.) to confirm the results, and PBS solution was used as a control. After verification, the biofilm samples were prepared in a cell flask, and the biofilm was scraped with a cell scraper. The biofilm was frozen with liquid nitrogen and subjected to omics analysis.

### Biomass and biofilm bacterial assays

The biomass assay was performed using crystal violet, and the viable bacteria were counted by trypticase soy agar (TSA) plates as described previously [20]. Briefly, the supernatant was discarded, and the cells were washed twice with sterile PBS. Fixation was carried out with 99% methanol for 10 min, followed by air drying, staining with 0.04% crystal violet solution for 20 min, and washing with sterile PBS. Then, 33% acetic acid was used to dissolve the bound crystal violet, and absorbance was measured at *OD*_600_ nm. To count the number of bacteria in the biofilm, 100 µL Triton-100X was added to each well to disrupt the biofilm, followed by 10-fold dilution and spreading on TSA plates, and the colonies were counted after 12 h at 37 ℃.

### CLSM

The morphological features of biofilms were observed by CLSM as described previously with some modifications [19]. In this experiment, 250 µL of the undiluted test bacterial solution (*OD*_600_ = 0.1) was added to an 8-well chambered cover glass (1.5 Borosilicate glass, Lab-Tek II chambered coverglass, Rochester, NY, USA), and the medium was replaced with fresh medium every 24 h. After incubation for 3 days at 37 ℃, the biofilm was treated with CRAMP at 37 ℃ for 1 h. Then, the biofilm was washed with 0.9% (wt/vol) NaCl and stained for 20 min in the dark at room temperature using a Filmtracer™ LIVE/DEAD™ Biofilm Viability kit (Cat. No. L10316; Molecular Probes, Thermo Fisher Scientific, Waltham, MA, USA).

After being rinsed with sterile water, the biofilm samples were imaged with a point-scanning confocal microscope (LSM-800; Zeiss GmbH, Oberkochen, Germany), which was equipped with a Plan-Apochromat 63×/1.40 oil objective lens. Signals were recorded using the green (SYTO9, excitation of 488 nm) and red (PI, excitation of 561 nm) channels. The three-dimensional (3D) image was constructed by stacking multiple images with different Z values (z-stack). The images were acquired using ZEN (black edition) software. Four representative images were selected from each biofilm, and each experiment was repeated at least three times. The biofilm volume, area and fluorescence intensity were analysed by BiofilmQ software [21].

### Transcriptomic assay

The PAO1 biofilm samples were analysed three times in a cell culture flask as described above. The biofilm samples were washed twice with the same volume of PBS solution and scraped with a cell scraper at 4 ℃. Then, the samples were stored at −80 ℃ after being quickly frozen with liquid nitrogen. The transcriptomic analysis (Hiseq4000; Illumina Inc., Chicago, IL, USA), quantitative real-time polymerase chain reaction (qRT‒PCR), and statistical analysis (*P* < 0.05, |log_2_FoldChange| >1) details are provided in Additional file 1. The primers used for qRT‒PCR were designed by Invitrogen Inc. (Carlsbad, CA, USA) and are listed in Additional file 1B.

### Proteomics analysis

The PAO1 biofilm samples were analysed three times in a cell culture flask as described above. The biofilm samples were washed twice with the same volume of PBS solution and scraped with a cell scraper at 4 ℃. Then, the samples were stored at −80 ℃ after being quickly frozen with liquid nitrogen. The protein extraction, liquid chromatography‒mass spectrometry (LC‒MS), and statistical analysis (*P* < 0.05, |log_2_FoldChange| >1) details are provided in Additional file 1.

### Metabolomics analysis

The PAO1 biofilm samples were analysed three times in a cell culture flask as described above. The biofilm samples were washed twice with the same volume of PBS solution and scraped with a cell scraper at 4 ℃. Then, the samples were stored at −80 ℃ after being quickly frozen with liquid nitrogen. The metabolite extraction, LC‒MS, and statistical analysis (*P* < 0.05, |log_2_FoldChange| >1) details are provided in Additional file 1.

### Analysis of EPS, alginate (Alg), and rhamnolipid contents in biofilms

The PAO1 biofilm samples were cultured in 6-well plates as described above. The biofilms were treated with CRAMP at 37 ℃ for 1 h. The biofilm samples were washed twice with the same volume of PBS solution and scraped with a cell scraper. The number of biofilm bacteria in the CRAMP group and control group was normalized to the same level, and the control group was considered 100% for calculation. The content of EPS was determined by the phenol‒sulfuric acid method [22], and the content of Alg was quantified by the 1,3-dihydroxynaphthalene method [23] with some modifications (Additional file 1). It is noteworthy that rhamnolipid may be secreted into the supernatant. The biofilms were treated with CRAMP at 37 ℃ for 1 h, while the biofilm sample and supernatant were scraped with a cell scraper for testing as a whole sample without the PBS washing step, which was different from the abovementioned procedure. The content of rhamnolipid was determined by the orcinol-sulfuric acid method [24] with some modifications (Additional file 1).

### Analysis of c-di-GMP level and swimming motility

The c-di-GMP level was detected as previously described [25] with some modifications. As the *luxCDABE* gene is more appropriate as a reporting system for c-di-GMP [26], the *gfp* reporter was replaced with *luxCDABE* to build p*cdrA::lux* (Additional file 1). PAO1 containing the plasmid p*cdrA::lux* was used to form biofilms, which were treated with CRAMP as described above. The bacterial biofilms and dispersed cells were collected, and bioluminescence intensity was measured at *OD*_600_ nm.

The swimming motility of biofilm cells was assessed as previously described with some modifications. The PAO1 biofilm samples were cultured in 6-well plates as described above.

### Biofilm dispersion and reformation of dispersion cells and sensitivity analysis

The preformed biofilms were divided into control, 62.5 µg/mL, 31.2 µg/mL, and 15.6 µg/mL groups and treated at 37 ℃ for 1 h with prediluted drugs. Then, the dispersed cells in each well were transferred to a sterile 96-well plate, and the biofilms were washed with PBS solution. The biofilm cells were collected by Triton-100X. The dispersion cells and biofilm cells were diluted tenfold and spread on TSA plates; colonies were counted after 12 h at 37 ℃. At the same time, 100 µL of dispersed cells and planktonic bacteria were added to 96-well plates, and the reformation ability of biofilms was assessed by crystal violet after 14 h. The sensitivity of dispersion cells and planktonic bacteria was tested by exposure to CRAMP and CIP. Viable bacteria were counted on TSA plates after exposure for 0, 1, 2, 3, and 5 h.

### Statistical analysis

Statistical analysis was performed using GraphPad Prism 8.0 software (GraphPad Software Inc., San Diego, CA, USA). An unpaired t test (two-tailed) was used to calculate the statistical significance. Significant differences are indicated as *(*P* < 0.05), **(*P* < 0.01), ***(*P* < 0.001), and ****(*P* < 0.0001).

## Results

### The antibiofilm activity of CRAMP against preformed PAO1 biofilms

**Figure 1:**
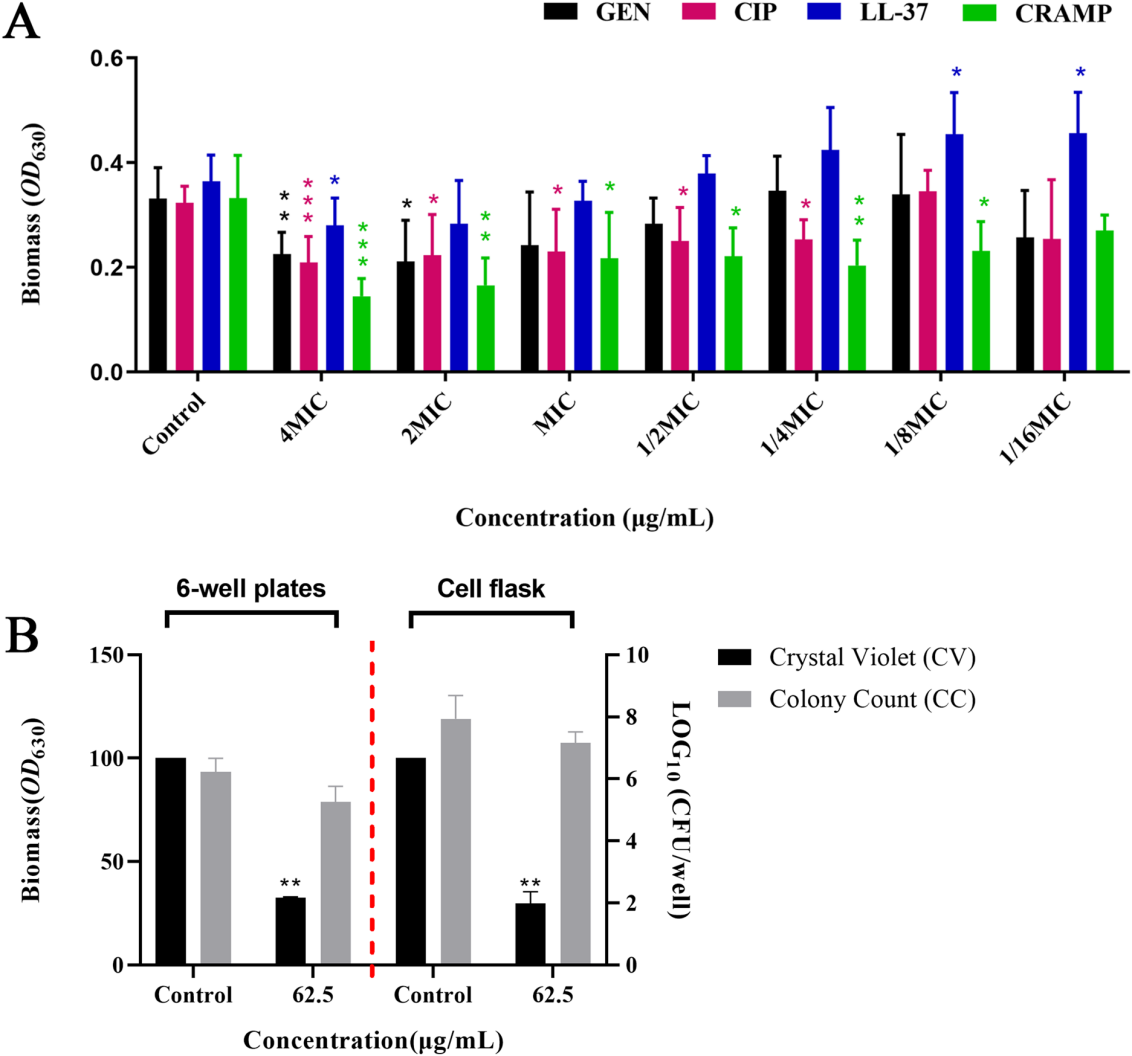
**Anti-biofilm activity of CRAMP against** ***P. aeruginosa*** **PAO1.** The PAO1 suspension was adjusted to *OD*_600_ = 0.1 with MH broth and diluted 100 times. The suspension was cultured in different culture plates at 37 °C and then treated with antibiotics or CRAMP for 1 h. The biomass was detected by crystal violet (CV) staining, and the viable bacteria of the biofilm were detected by colony counting (CC). **A** Represents the biomass of PAO1 biofilm (1 day) treated with CRAMP, LL-37, gentamicin (GEN), and ciprofloxacin (CIP) in 96-well plates detected by CV. **B** Represents the biomass and viable bacteria, in which the PAO1 biofilm (3 days) was treated with CRAMP in 6-well plates and cell culture flasks. An unpaired t test (two-tailed) was used to measure statistical significance. * *P* < 0.05, ** *P* < 0.01, *** *P* < 0.001 compared with the control group.

To investigate the antibiofilm activity of CRAMP against PAO1 biofilms, different concentrations of CRAMP were used on 1-day-old preformed biofilms. These concentrations were lower than the lowest cytotoxic concentration (data not shown). In addition, two common antibiotics (gentamicin and ciprofloxacin) and human AMP LL-37 were used as controls. The results showed that CRAMP exhibited a more significant effect on reducing the biomass of PAO1 biofilms, with a reduction rate of 56.6% at 4 MICs (62.5 µg/mL) (Figure 1A). After screening the optimal concentration, the antibiofilm activity of CRAMP was assessed in different culture systems. To form optimal biofilms for use in the omics data analysis, preformed biofilms from different time points were studied. The results revealed that 3-day-old preformed biofilms were the most stable biofilms and thus could be used for the whole study. Furthermore, CRAMP exhibited a significant eradicative effect on 3-day-old preformed biofilms at a concentration of 62.5 µg/mL (Figure 1B).

**Figure 2:**
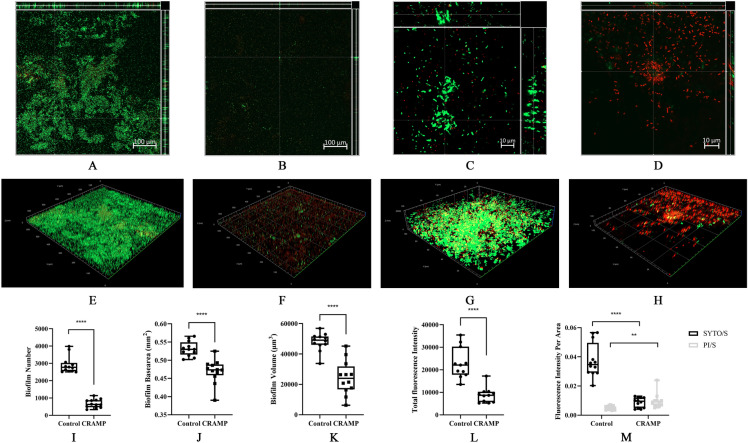
**Confocal laser scanning microscopy of the eradication of PAO1 biofilms treated with CRAMP.** The bacterial suspension was cultured in a chamber coverglass for 3 days, and the biofilm was treated with CRAMP for 1 h. Staining with SYTO 9 (green, live) and propidium iodide (red, dead) was performed under objective 20× and 63× oil by CLSM. The orthogonal views of Z-stacks and 3D images are presented (ZEN 2.3 Viewer v2.3.69.1000 software). Image processing was carried out using BiofilmQ software. **A** and **E** represent the orthogonal views and 3D biofilm representation in the objective of 20× in the control group. **B** and **F** represent the orthogonal views and 3D biofilm representation of CRAMP in the objective of 20×. **C** and **G** represent the orthogonal views and 3D biofilm representation of the objective of 63× in the control group. **D** and **H** represent the orthogonal views and 3D biofilm representation of CRAMP in the objective of 63×. **I** represents the number of biofilms treated with CRAMP. **J** represents the biofilm base area treated with CRAMP. **K** represents the volume of biofilms treated with CRAMP. **L** represents the fluorescence intensity of biofilms treated with CRAMP. **M** represents the ratio of fluorescence intensity to biofilm base area treated with CRAMP. An unpaired t test (two-tailed) was used to measure statistical significance. * *P* < 0.05, ** *P* < 0.01, **** *P* < 0.0001 compared with the control group.

To visualize the anti-biofilm activity of CRAMP, CLSM was applied after live (SYTO 9) and dead (PI) staining was performed with the tested biofilms. The results showed that at a concentration of 62.5 µg/mL, CRAMP markedly decreased PAO1 biofilms with a reduction in the number (76.41%), area (11.62%), and volume (49.39%) of biofilms (Figures 2I–K). Under CLSM (Figure 2), the effect of CRAMP on *P. aeruginosa* biofilms was similar to previously reported findings [20]. Furthermore, the total fluorescence intensity of living (SYTO 9) and dead (PI) bacteria was reduced to 61.9% (Figure 2L). In addition, the ratio of fluorescence intensity to biofilm surface per unit area decreased after treating the biofilms with CRAMP. It is noteworthy that the live bacteria per unit area of CRAMP decreased by 4.31 times, while the dead bacteria per unit area was elevated by 1.99 times compared with that of the control (Figure 2M).

### CRAMP inhibited the synthesis of polysaccharides

The results of the omics data analysis are presented in Additional file 1F. The transcriptome sequencing data were consistent with the qRT‒PCR results (Additional file 1F). A hierarchical clustered heatmap indicated the differentially expressed genes (DEGs) and differentially expressed proteins (DEPs) of PAO1 biofilm cells after CRAMP treatment (Additional file 1F). Consistently, score plots of unsupervised principal component analysis showed that the metabolic profiles of PAO1 biofilm cells were significantly different after CRAMP treatment (Additional file 1F). The fold changes of totally different genes, proteins, and metabolites in the omics data analysis are shown in Additional files 2, 3, 4. The DEGs and DEPs in regulatory pathways that are associated with biofilms are separately presented in two Figures (Additional files 1G, H).

**Figure 3:**
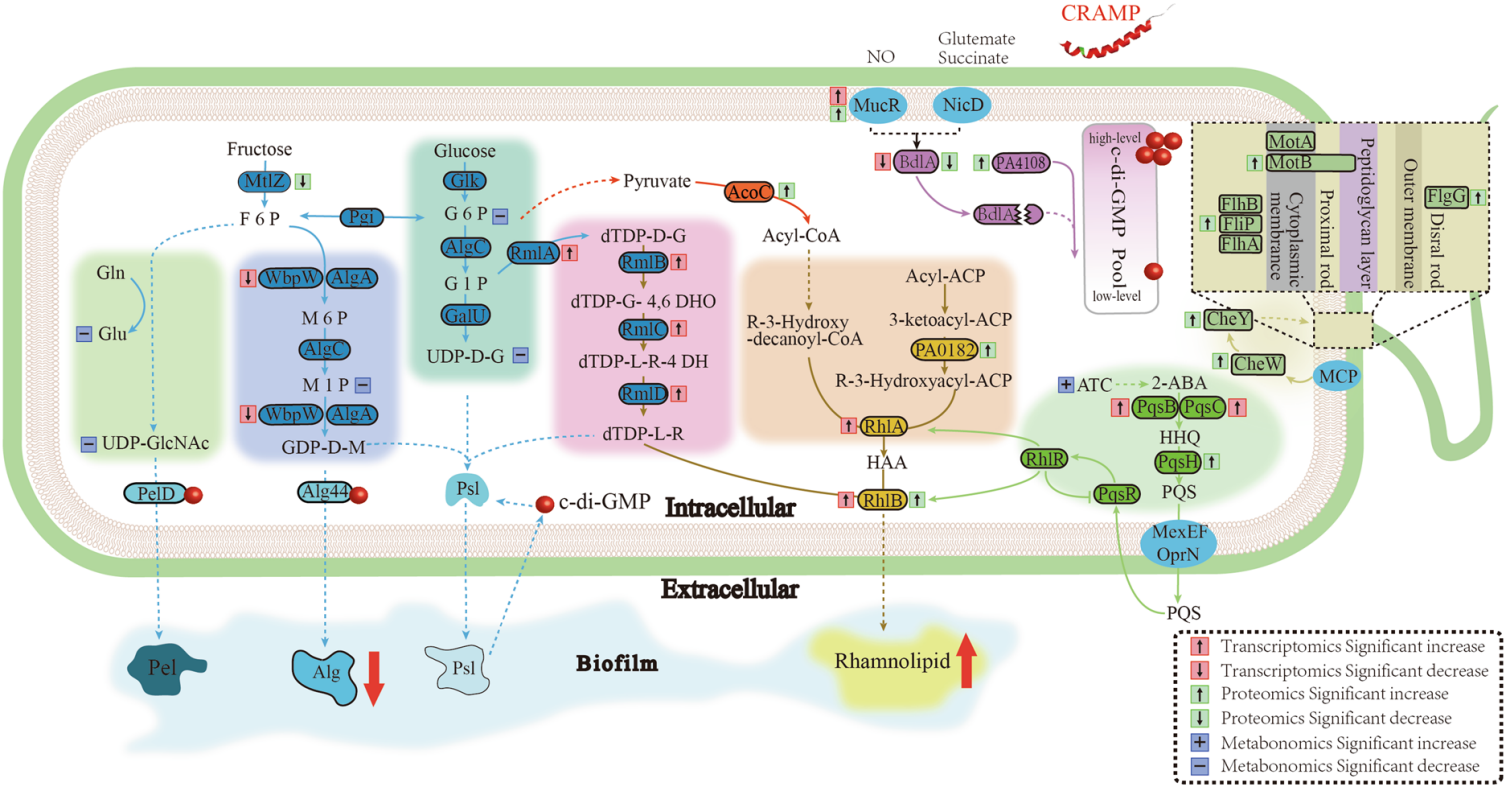
**Schematic diagram of the enriched regulatory pathways in** ***P. aeruginosa*** **PAO1 biofilms treated with CRAMP based on transcriptomics, proteomics, and metabolomics analyses.** The preformed PAO1 biofilm (3 days) was treated with 62.5 µg/mL CRAMP for 1 h. CRAMP mainly involved the synthesis of exopolysaccharide, c-di-GMP metabolism, regulation of the PQS system, and flagellar assembly. The red dots represent c-di-GMP.

**Figure 4:**
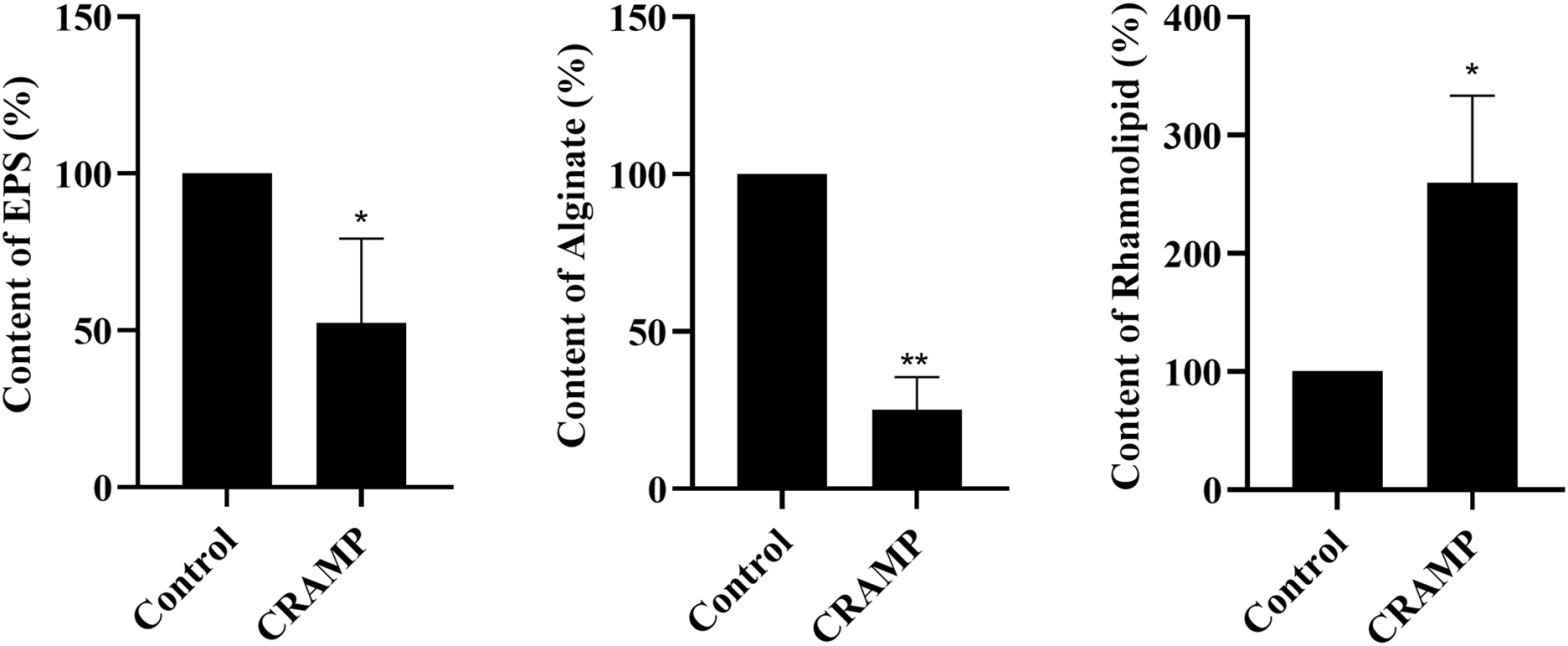
**The contents of EPS, alginate, and rhamnolipid in** ***P. aeruginosa*** **PAO1 biofilms treated with CRAMP.** The bacterial suspension was cultured in 6-well plates for 3 days, and the biofilm was treated with CRAMP for 1 h. The biofilm was collected with a cell scraper, and the contents of EPS (total polysaccharides of biofilms + total protein of biofilms), alginate, and rhamnolipid in the biofilms were detected. **A**–**C** represent the percentages of EPS, alginate, and rhamnolipid in the PAO1 biofilms treated with CRAMP, respectively. The number of biofilm bacteria in the CRAMP and control groups was normalized to the same level, and the control group was considered 100% for calculation. An unpaired t test (two-tailed) was used to measure statistical significance. *P < 0.05, **P < 0.01 compared with the control group.

Crystal violet assay and CLSM observations showed that the PAO1 biofilms were notably changed after CRAMP treatment. *Pseudomonas aeruginosa* mainly produces three EPS, Alg, Psl, and Pel, which play a critical role in biofilm formation [27]; thus, we searched for changes in gene expression levels related to EPS synthesis in the omics data. Unexpectedly, no direct evidence was found that CRAMP could inhibit EPS synthesis in 2914 DEGs and 785 DEPs (Additional files 2, 3), while transcriptional regulators were not included. Only the expression level of PslI (a glycosyltransferase) was significantly downregulated (Additional file 1H). However, 280 differential metabolites were included, providing valuable information (Additional file 4). The expression levels of uridine diphosphate N-acetylglucosamine (UDP-GlcNAc) and UDP glucose dehydrogenase (UDP-D-G), which are the synthetic precursors of Pel and Psl, respectively, were markedly lower than those in the control group (Figure 3) [28, 29]. The same results were observed in d-glucose 6-phosphate (G6P) and d-mannose 1-phosphate (M1P), in which G6P was converted to UDP-D-G via AlgC and GalU or via glycolysis to participate in the tricarboxylic acid cycle (TCA) (Figure 3) [28]. M1P can be converted into GDP-D-M through WbpW or AlgA to contribute to the synthesis of Alg or Psl (Figure 3) [28]. These data indicated that exopolysaccharide secretion was insufficient. According to the omics data, the levels of EPS and Alg in PAO1 biofilms were detected by phenol‒sulfuric acid and 1,3-dihydroxynaphthalene colorimetric methods, respectively [22, 23]. These results showed that the levels of EPS (*P* < 0.05) and Alg (*P* < 0.01) in the PAO1 biofilms were markedly reduced after treatment with CRAMP (Figures 4A and B).

It has been reported that rhamnolipid synthesis can inhibit the production of EPS [30], suggesting that the reduced glucose precursors may be utilized in rhamnolipid synthesis. Transcriptomics showed that the expression levels of dTDP-L-rhamnose-related genes (*rmlA*, *rmlB*, *rmlC*, and *rmlD*) were upregulated (*P* < 0.01), and the expression level of *rhlA*, which is involved in the synthesis of R-3-((R-3-hydroxyalkanoyl) oxy) alkanoic acids (HAAs), was elevated (Additional file 1G) (Figure 3) [28, 31]. It is noteworthy that the gene and protein expression levels of RhlB, which links rhamnose and lipid chains together to form mono-rhamnolipid, were significantly altered (Additional file 1H) (Figure 3). On the other hand, the *Pseudomonas* quinolone signal (PQS) system, as a self-induced quorum sensing system of *P. aeruginosa*, can influence a variety of bacterial virulence factors (including rhamnolipids) and biofilm formation [5]. The expression levels of genes or proteins related to the synthesis of the PQS system were measured, of which the expression levels of *pqsB*, *pqsC* (Additional file 1G), PqsH (Additional file 1H), and *mexEF-oprN* (Additional file 2) of efflux pumps involved in the secretion of PQS were significantly upregulated (Figure 3) [5, 32]. These findings suggested that the rhamnolipid content of the PAO1 biofilm could increase after CRAMP treatment (Figure 3). Subsequently, it was revealed that the rhamnolipid content of biofilms significantly increased (*P* < 0.05) (Figure 4C).

### CRAMP induced dispersion of biofilm cells

**Figure 5:**
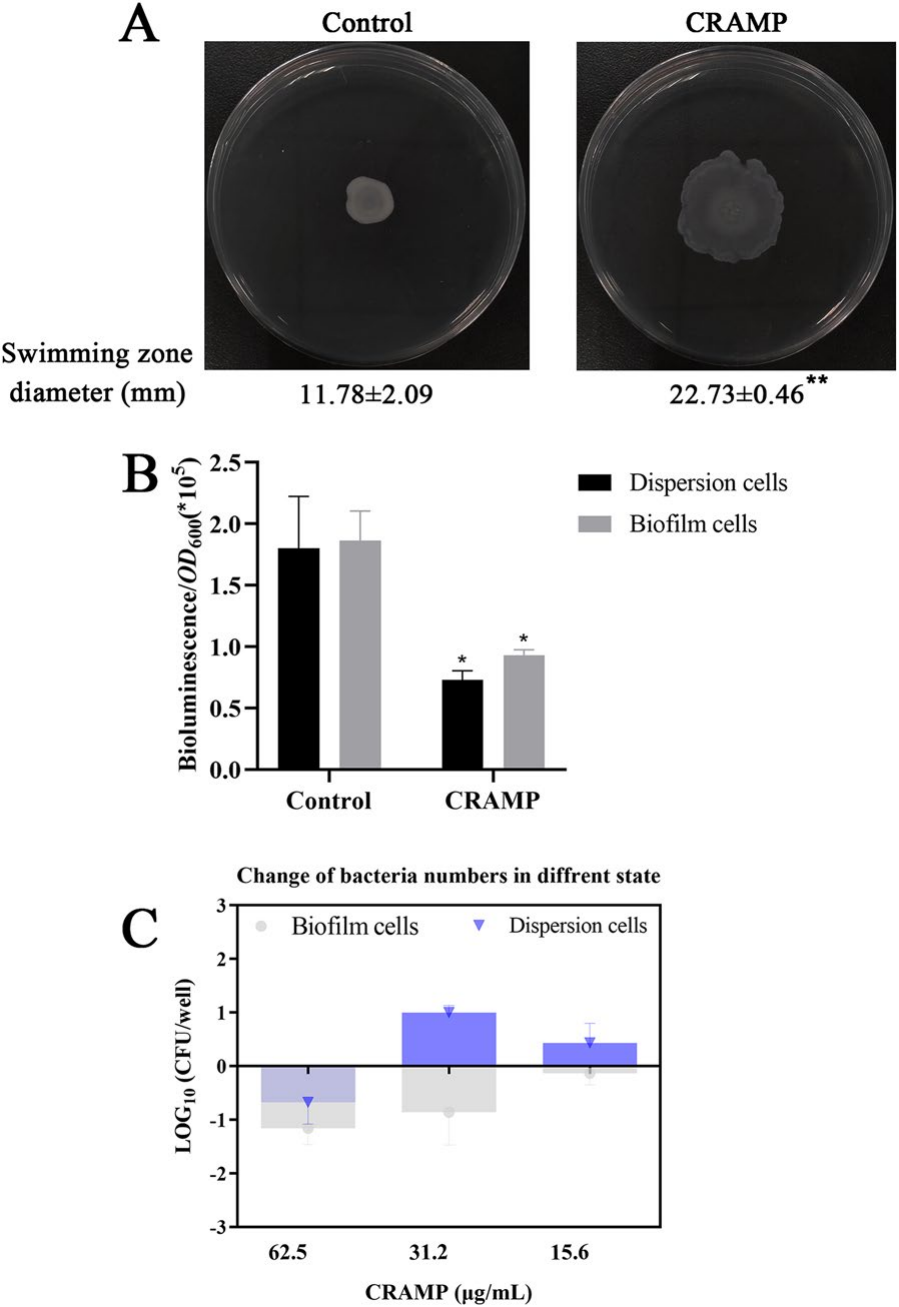
**Swimming motility and c-di-GMP level of bacterial biofilms treated with CRAMP.** **A** represents visual comparison and quantitative determination of the swimming motility of PAO1 biofilms (3 days) treated with CRAMP. Dispersal cells were inoculated into the plates with 0.5% agar and grown for 24 h. Swimming motility was measured by diffusion diameter on the agar surface. **B** represents the c-di-GMP level of PAO1 biofilms treated with CRAMP measured using the p*cdrA::lux* reporter. Bioluminescence and OD were measured using a plate reader. The c-di-GMP level was calculated as Lux/OD600. **C** represents the change in log_10_ of PAO1 in different states (biofilm cells or dispersion cells) compared with the control group after treatment with different concentrations of CRAMP. The blue colour represents the change in the number of dispersed cells in the upper suspension compared with that of the control group, and the grey colour represents the change in the number of biofilm cells compared with that of the control group. An unpaired t test (two-tailed) was used to measure statistical significance. *P < 0.05, **P < 0.01 compared with the control group.

The dispersion of biofilms is directly associated with the c-di-GMP level of bacterial biofilms. Significant changes in the expression levels of *amrZ* and *tpbA* were found in the transcriptomic data (Additional file 1G). The expression levels of AmrZ [33] and Tpb [34, 35] are correlated with c-di-GMP levels. In addition, c-di-GMP is synthesized by diguanylate cyclases (DGCs) and degraded by phosphodiesterases (PDEs) [36]. Importantly, there were no changes in GGDEF domain proteins, while the expression level of PA4108 (containing HD-GYP domains) was significantly upregulated. In contrast, the expression level of cyclic di-GMP (c-di-GMP) phosphodiesterase PA4781 is significantly reduced (Additional file 1G) (Figure 3). In addition, the dispersion of biofilms is affected by a variety of environmental factors [37]. While MucR (markedly upregulated in the present study, Additional file 1G), NbdA, and NicD are important environmental sensing proteins located in the inner membrane [1]. The BdlA protein (which was markedly downregulated in the present study, Additional file 1G) (Figure 3) plays a role in mediating biofilm dispersion in response to environmental factors; however, the BdlA protein is active only when cleaved into fragments [38]. The abovementioned results indicated that the c-di-GMP level in PAO1 biofilm cells significantly decreased after treatment with CRAMP. Therefore, the c-di-GMP level in biofilm cells and dispersed cells was detected by introducing the p*cdrA::Lux* plasmid into the PAO1 strain, and it was found that the c-di-GMP level was reduced by half after treatment with CRAMP compared to the control group (Figure 5B).

Studies have demonstrated that biofilm formation might reduce bacterial motility via an increase in c-di-GMP levels, while biofilm dispersion would result from improved bacterial chemotaxis due to the reduced level of c-di-GMP [1, 11]. Proteins in the membrane that are related to the bacterial chemotaxis system, such as putative chemotaxis transducer Cttp, probable chemotaxis transducer PA1251, and methyl-accepting chemotaxis protein PctC, were significantly changed at the transcriptional level or protein level (Additional file 1H). The expression levels of chemotaxis protein CheW (wspd and PA0177) and Fis family transcriptional regulator CheY (regulating the level of chemotaxis protein PA0179), which links environmental information to the flagellar assembly system to enhance bacterial motility, were significantly upregulated (Figure 3). Furthermore, the expression levels of several proteins in the flagellar assembly system were also elevated (Additional file 1H). In subsequent phenotypic validation tests, semisolid AGAR was used, and it was found that the bacterial motility of the PAO1 biofilms was significantly enhanced after CRAMP treatment (Figure 5A).

The omics data suggested that CRAMP could disperse the PAO1 biofilms. To further confirm this, after treatment with CRAMP for 1 h, the upper-chamber suspension cells and the biofilm cells in the PAO1 biofilms were observed under the lower concentration (Figure 5C). At concentrations of 62.5, 31.25, and 15.6 µg/mL, the number of biofilm cells was reduced by 93.1%, 86.2%, and 25.9%, respectively. Additionally, the dispersed cells showed a 79.1% decrease compared with that in the control group at a concentration of 62.5 µg/mL (further experiments confirmed that this concentration of CRAMP could kill some dispersed cells). Nonetheless, the number of dispersed cells was elevated by 90.0% and 63.7% after treatment with CRAMP at concentrations of 31.5 and 15.625 µg/mL, respectively, which was almost consistent with the decrease in bacterial biofilms.

### Phenotypes of dispersed cells

**Figure 6:**
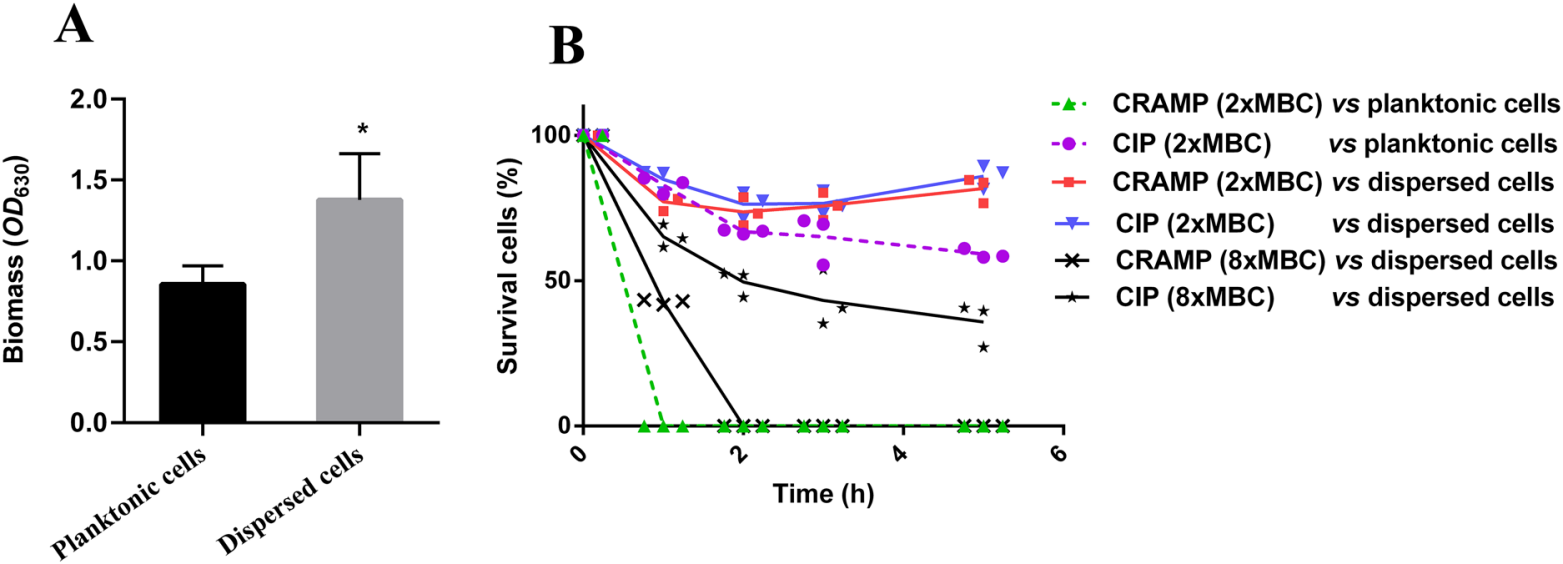
**Testing the biofilm reformation ability and antibiotic sensitivity of dispersed cells.** CRAMP was used to disperse the biofilm and was preformed for 3 days, and the dispersed cells were collected for testing, with planktonic cells as a control. **A** represents the biofilm reformation capacity of dispersed cells and planktonic cells within 14 h, and the biomass was detected by crystal violet staining. **B** represents the antibiotic sensitivity of dispersion cells and planktonic cells, the Y-axis represents the survival rate of dispersion cells and planktonic cells, and the X-axis represents the exposure time to drugs. The dotted line represents planktonic cells, and the solid line represents dispersed cells. Black filled triangle represents planktonic cells that were treated with CRAMP (2 MBCs, 62.5 µg/mL); Black filled circle represents planktonic cells that were treated with ciprofloxacin (2 MBCs, 0.125 µg/mL); Black filled square represents dispersed cells that were treated with CRAMP (2 MBCs, 62.5 µg/mL); Black filled inverted triangle represents dispersed cells that were treated with ciprofloxacin (2 MBCs, 0.125 µg/mL). × represents dispersed cells that were treated with CRAMP (8 MBCs, 250 µg/mL); Black filled star represents dispersed cells that were treated with ciprofloxacin (8 MBCs, 0.5 µg/mL). An unpaired t test (two-tailed) was used to measure statistical significance. *P < 0.05, **P < 0.01.

Changes in the expression levels of bacterial virulence factor-related genes were also noted in the analysis of the omics data, in which the expression levels of extracellular enzymes (*exoT/exoS*) (Additional file 2) and alkaline proteases (*aprF*, *aprA*, and *aprD*) were significantly reduced (Additional file 2). However, the expression levels of alkaline phosphatase *eddA* (Additional file 2) and three genes related to hydrocyanic acid production (*hcnA*, *hcnB*, and *hcnC*) (Additional file 2) were significantly upregulated. Additionally, significant changes were found in the multidrug efflux pumps (Additional file 3), in which significant upregulation in several multidrug efflux systems (e.g., MexCD-OprJ, MexAB-OprM, etc.) was observed. In particular, the protein level of MxeCD-OprJ was also significantly elevated (Additional file 3). Thus, we attempted to analyse the biofilm reformation ability and antibiotic sensitivity of dispersed cells. The results of crystal violet staining showed that the ability of dispersed cells to reform biofilms was significantly more potent than that of planktonic cells (Figure 6A). In addition, under CRAMP exposure of 2 MBCs, after 1–5 h, the survival rate of planktonic cells dropped to 0% (Figure 6B). However, the dispersed cells that were exposed to both ciprofloxacin and CRAMP of 2 MBCs were still alive at 4–5 log_10_ CFU/mL after 1–5 h; thus, the concentration of CRAMP increased, and the study continued. When the dispersed cells were exposed to CRAMP (8 MBCs) for 2, 3 and 5 h, the survival rate of dispersed cells was reduced to 0%. However, when the dispersed cells were exposed to ciprofloxacin (8 MBCs) for 2, 3 and 5 h, the survival rates of dispersed cells were 49.52%, 43.22%, and 35.76%, respectively (Figure 6B). The abovementioned results indicated that the sensitivity of dispersed cells to antibacterial drugs decreased compared with that of planktonic cells, and the ability of CRAMP to reduce the number of dispersed cells was superior to that of ciprofloxacin at 8 MBCs within the time range that the dispersed cells maintain their phenotype. Previous studies reported that dispersed cells were grown in a medium with a dispersal agent to maintain their phenotype for 5 h [12, 39].

## Discussion

The present study used 96-well plates to screen the optimal intervention conditions for CRAMP. Due to the large amount of biofilm needed for the omics samples, we tried different culture systems and found that cell culture flasks could meet the experimental needs. In addition, later phenotypic verification (such as the determination of rhamnolipids, etc.) mainly used 6-well plates, so we verified the phenotype of eradicating biofilms by CRAMP in cell culture flasks and 6-well plates.

Integrative analysis of transcriptomic, proteomic, and metabolomic data has been widely used to study biofilms in recent years [40–42]. Preliminary experiments found that the biofilms treated with CRAMP at a concentration of 62.5 µg/mL for 1 h exhibited the best eradication effect, and the changes in the transcription levels of biofilm-related genes were evident under this experimental condition. Therefore, we finally adopted this condition to prepare the samples of the CRAMP group. It is generally accepted that the reduced volume and number of biofilms can be mainly accompanied by decreased levels of EPS. According to the omics data, we first targeted the pathway of PAO1 biofilm exopolysaccharide synthesis. The exopolysaccharide of *P. aeruginosa* is mainly categorized into the following types: Pel, Psl, and Alg [43]. The establishment of solid surface-associated biofilms is greatly dependent on the presence of Pel [1, 44]. Psl and Alg play an important role in stabilizing the 3D structure of biofilms [1, 44]. EPS can inhibit chemotaxis of neutrophils and reduce antibiotic penetration [45, 46]. Therefore, reducing the synthesis of EPS can not only delay the formation of biofilms but also reduce stimulation of the host body [45, 46]. In addition, rhamnolipid, as a biosurfactant, is involved in the process of dispersing bacterial biofilms and contributes to the disassembly of biofilms [47–49]. Additionally, rhamnolipid synthesis competes with the precursors necessary for the synthesis of EPS [30], reducing the production of extracellular matrix and creating conditions for the dispersion process. In the present study, the phenotypic validation experiments demonstrated that CRAMP increased the content of rhamnolipid while reducing the levels of EPS, especially Alg.

It has been found that low intracellular c-di-GMP content induced the expression levels of the QS systems, which led to the increased production of rhamnolipid [50]. In the present study, a decrease was found in intracellular c-di-GMP levels in the PAO1 biofilms treated with CRAMP using the p*cdrA::lux* reporting system. The decrease in c-di-GMP levels in biofilm cells is an important feature of biofilm dispersion [1, 11]. In a study of the effects of NO on biofilm dispersion, the environmental sensing role of intermembrane proteins (MucR and NbdA) was found [51]. A signal is sent to phosphodiesterase (PDEs) and diguanylate cyclase (DGCs) by cleavage of BdlA protein to regulate the c-di-GMP level in cells, leading to the modulation of biofilm dispersion [38, 52]. This signal transduction pathway is associated with the interaction between BdlA and PDE DipA, resulting in an increase in DipA levels, elevation of PDE activity, and a decrease in c-di-GMP levels [38]. It is noteworthy that no change in PDE DipA was observed in both our transcriptomic and proteomic data. However, the protein levels of PA4108, which has a PDE domain, were significantly increased. We speculate that the interaction of BdlA protein with PA4108 (rather than DipA) in PAO1 biofilms treated with CRAMP causes an increase in the activity of PDEs, causing a reduction in the c-di-GMP level. The protein level of another protein, PA4781, which contains the HD-GYP domain of PDEs, was significantly upregulated, which might be caused by a negative feedback mechanism with the increase in PA4108 levels.

In research on the effects of nutrients on biofilm dispersion, it was reported that flagellum-driven motility could be a major mechanism of release [53]. In the present study, the motility of the dispersed bacterial flagella released after treatment with CRAMP was investigated, and it was noted that the motility was significantly enhanced. Importantly, the synthesis of EPS, in addition to being directly associated with the expression levels of polysaccharide synthetic genes or proteins, can be regulated by the c-di-GMP level. Alg 44 and Pel D regulated Alg and Pel production in PAO1 biofilms by binding with c-di-GMP [54, 55]. A high c-di-GMP level relieves transcriptional repression at the psl promoters by binding to the regulator FleQ and then leads to the increased production of Psl, which is a positive feedback regulatory mechanism [56]. c-di-GMP-binding effector proteins are involved in the synthesis of the three exopolysaccharides, Alg44 [54], PelD [55], PilZ [57], and FleQ [57], in which c-di-GMP positively regulates the synthesis of the three exopolysaccharides. This is consistent with our observation of a reduction in the levels of EPS and c-di-GMP in PAO1 biofilms treated with CRAMP. It was reported that a low c-di-GMP level in *P. aeruginosa* cells increased the production of rhamnolipid (a pqs and rhl-regulated virulence factor), and the induction of pqs and rhl QS expression requires the transcriptional regulator PqsR [50]. The results of the present study also showed a decrease in c-di-GMP levels and an increase in rhamnolipid levels; thus, we speculated that c-di-GMP-binding effector proteins should also be in the rhamnolipid synthesis pathway. Rhamnolipid synthesis is regulated by the PQS system; thus, crosstalk occurs between c-di-GMP and the PQS signalling pathway, and this key connection point may be PqsR and RhlR, which may also be the c-di-GMP-binding effector proteins. The regulation of c-di-GMP in this pathway occurs with a negative feedback mechanism. Therefore, the process of CARMP-induced PAO1 biofilm dispersion could be summarized as follows: Cleavage of BdlA activated PA4108 through the action of CRAMP, resulting in a decrease in c-di-GMP levels. A low c-di-GMP level could trigger a series of phenotypic changes, including enhanced bacterial flagellar motility and reduced synthesis of EPS (which is regulated by binding to the corresponding c-di-GMP effector protein). In addition, a low c-di-GMP level regulated the increased synthesis of rhamnolipid through the PQS system, which assisted in the dispersal of biofilms, including triggering the shedding of biofilms and competitively inhibiting the production of EPS.

To date, several studies have concentrated on the inhibition of biofilm formation, while bacterial dispersion needs to be further studied [1]. Dispersed cells are characterized by distinct gene expression and protein production patterns, as well as increased susceptibility to antimicrobial agents compared with that of their sessile counterparts [1, 11]. Although biofilm dispersion is a promising method to control biofilm infections, new problems have arisen, such as increased virulence and enhanced adhesion, etc., in some dispersed bacteria, which are worthy of additional investigation. The transcriptomic data of the present study suggested that enhanced bacterial invasiveness may occur by the increase in virulence factors after the dispersion of biofilm cells, which is consistent with the results of a previous study [58]. Additionally, the dispersed cells colonize new places with high mobility and form new biofilms [1]. The results of the current study showed that dispersed cells have a strong ability to form biofilms and compared to planktonic cells, are more drug-resistant. This suggests that the presence of biofilm dispersants may increase the risk of infection in clinical practice. In recent years, the use of dispersants combined with antibiotics has been reported, and this combination is highly advantageous [59–61]. In our previous study, it was shown that the combination of subinhibitory concentrations of CRAMP and colistin has a significant synergistic effect on the formation of PAO1 biofilms [19], and we also recently found that CRAMP combined with vancomycin, roxithromycin, and azithromycin showed faster and stronger antibiofilm bacterial activity than that of a single drug through the time-kill curve test, especially combination with vancomycin, which caused the biofilm cells to die within 3 h (a manuscript is in the preparation stage). These studies demonstrate that it is promising to develop CRAMP as a potential biofilm dispersant.

In addition to anti-biofilm activity, another study showed that CRAMP enhanced gut homeostasis as an immunomodulatory treatment for infectious colitis in pigs, and CRAMP showed no adverse side effects [62]. Although CRAMP exerts its immunomodulatory function to protect the host against microbial infection, it is still necessary to explore the efficacy of CRAMP against biofilms derived from different bacteria that cause infection in animals. In brief, CRAMP has the potential to be developed as a new antibacterial therapeutic to prevent and control biofilm-associated infections in humans or animals in the future.

In conclusion, the present study showed that CRAMP reduced the c-di-GMP level in PAO1 biofilm cells, triggering a decrease in EPS, especially Alg, and an increase in bacterial flagellar motility and rhamnolipids, contributing to the dispersal of biofilms. Therefore, CRAMP can be further studied and developed as a potential biofilm dispersant.

## Supplementary Information

**Additional file 1. Detailed methods or results covered in this article.**

**Additional file 2. Differentially expressed gene data.**

**Additional file 3. Differentially expressed protein data.**

**Additional file 4. Differentially metabolic substance data.**

## Data Availability

The raw RNA-seq datasets generated during this study are available through NCBI’s BioProject database under accession number PRJNA816204. The raw proteomics and metabolomics data are available upon request. The authors declare that all other relevant data supporting the claims of the paper are available either in the main text or Additional file.
