# Supplementary material for: Pseudomonas aeruginosa biofilm dispersion by the mouse antimicrobial peptide CRAMP
Source: Vet Res. 2022 Oct 8;53:80. doi: 10.1186/s13567-022-01097-y (PMC9548163; doi:10.1186/s13567-022-01097-y)
Supplement: Supplementary file 1 — Additional file 1. Detailed methods or results covered in this article. [file 13567_2022_1097_MOESM1_ESM.docx]

**Transcriptomic Assay**

The total RNA was isolated using a Total RNA extraction reagent for TRNzol (Tiangen Biotech). The concentration and quality were determined using a NanoDrop spectrophotometer (Implen) and Agilent 2100 Bioanalyzer (Agilent Technologies). The cDNA library was constructed using the following methods. Briefly, Fragmentation buffer was added to cleave the mRNA into short fragments. First strand cDNA was synthesized by random hexamer primers using mRNA as template. Double-stranded cDNA was subsequently synthesized with dUTP incorporation into the second strand. AMPure XP beads system was used to purify and select double-stranded cDNA. After the mRNA was purified and fragmented, sequencing using the Illumina Hiseq4000 (Illumina).

Raw reads were processed by Trimmomatic software v0.33 to obtain clean reads by trimming the low-quality Reads and adapter sequences. The screening conditions as follows: (a) reads with adaptor sequences, (b) reads with N (N represented unknown bases) account for more than 1% of the entire read length, and (c) low quality reads (where the number of bases with Q ≤ 20 accounts for more than 50% of the entire read length). Then, Data was aligned to the reference genome (*Pseudomonas aeruginosa* PAO1 complete genome, GenBank: AE004091.2) using Bowtie2 software. Gene expression level was values by Fragments per kilobase of transcript per million mapped reads (FPKM). The differentially expressed genes (DEGs) identified by both the fold change (|log2FoldChange|>1) and the *p*-value (< 0.05).

**Quantitative Real-time Polymerase Chain Reaction (qRT-PCR)**

Fifteen DECs from the RNASeq analysis was examined by qRT-PCR to confirm the RNA-Seq results. The qRT-PCRs were performed with RNA samples used for RNAseq. PrimeScript™ RT Reagent Kit with gDNA Eraser (TaKaRa) was used for cDNA reverse transcription. Quantitative real-time PCR was performed on a ABI7500 Real-Time PCR Detection System (Applied Biosystems) using SYBR® Premix Ex Taq™ II (Tli RNaseH Plus), ROX plus (TaKaRa) according to the manufacturer’s instructions. Gene-specific primers were shown in the Supplementary Table 2, and the 16S rRNA gene was used as the internal control. PCR was performed according to the following steps: 30 s at 95 ℃, 40 cycles of 5 s at 95 ℃, and 40 s at 60 ℃. Melting curve analysis of amplification products was performed to evaluate the specificity of the amplification. The relative expression was calculated using the 2^-△△Ct^ method. Samples were run in triplicate, and the experiments were repeated at least three times.

**Proteomics analysis**

The biofilm sample Add 800μL protein extraction buffer (500mM Tris-HCl buffer, 50mM EDTA, 700mM Sucrose, 100 mM KCl solution, 2% β -mercaptoethanol and 1 mM phenyl sulfonyl fluoride, pH adjusts to 8.0 with HCl) into the grinding tube, Mixed, grinding for 10 min. Then, the same volume of Tris-phenol was added, and the grinding was continued for 10min. After centrifugation at 5500 *g* at 4 ℃ for 10 min, Add 4 times of the volume of cold acetone to the supernatant over night at −20 ℃. After centrifugation at 5500 *g* at 4 ℃ for 10 min, the obtained precipitate was washed with cold acetone three times, air dry. Four percent SDS was use to redissolve protein precipitates. The total protein extracted from the samples was quantitatively analyzed by BCA (Bicinchoninic acid) (Solarbio) method, according to the manufacturer’s protocol. The FASP methods was use to digest the protein with trypsin, Then, C18 Cartridge was used to desalinate the peptide. After lyophilization, the peptide was redissolved with 40 μL 0.1% formic acid solution, and the peptide was quantified (*OD*_280_).

For liquid chromatography mass spectrometry (LC-MS), LC (Q Exactive, Thermo Fisher Scientific)-MS (EASY-nLC, Thermo Fisher Scientific) equipped with a reversed-phase pre-column (Acclaim PepMap 100, Thermo Fisher Scientific) and separation by Easy column (75μm x 15cm, 3 μm, Thermo Fisher Scientific). Solvent A is 0.1% Formic acid in water, solvent B is 0.1% Formic acid in Acetonitrile. The chromatographic separation conditions were as follow: peptides were dissolved in solvent A; flow rate: 400 nL/min; injection volume: 5 μL. The peptides were subjected to nanoelectrospray ionization and mass spectrometry conditions were as follow: MS ion scan range: 300-1800 m/z, resolution: 70000-200 m/z; Collision Energy (NCE): 27%; resolution of MS/MS: 17500-200 m/z.

Proteome Discoverer 2.1.0182 (Thermo Fisher Scientific, Rockford, IL, USA) was used to process raw data files. Detailed setting of data processing parameters was shown in the Additional file 1C. Data were aligned with Uniprot *Pseudomonas aeruginosa* data (TaxID: 208964). The mapDIA software, based on Bayesian model, was used to differential protein screening for quantitative protein data to ensuring results credible. The differentially Proteins identified by both the fold change (|log_2_FoldChange|>1) and the *p*-value (< 0.05).

**Metabolomics analysis**

The 20 mg of biofilm sample was mixed with 500 μL solution (Methanol: Acetonitrile: Water=2:2:1) and vortex for 30 s. the mixture was subject to grind at 60 Hz for 120 s, and sonication for 10 min, stored at -20 ℃ for 1 h. After 13 000 rpm/min centrifugation for 15 min at 4 ℃, The four hundred μL of supernatant was lyophilizationed. One hundred μL of solution (Acetonitrile: Water=1:1) was added to lyophilization product, vortex for 30 seconds and sonication for 10 min. After 13000 rpm/min centrifugation for 15 min at 4 ℃. finally, the supernatant to following analyses.

For liquid chromatography mass spectrometry (LC-MS), LC-MS (AB Sciex, AB SCIEX Triple TOF 5600+) equipped with a Waters ACQUITY UPLC Amide (2.1 x 100 mm, 1.7 μm) was used. The chromatographic separation conditions were as follow: column temperature: 40 °C; flow rate: 0.5 mL/min; injection volume: 2 μL. The mass spectrometry conditions were as follow: heater temperature (TEM) 650 °C; Positive mode (ESI+) electrospray voltage: 5.5 KV; Negative mode (ESI-) electrospray voltage: 4.5 KV; Declustering potential: 60 V; Ion Source Gas1: 60 psi; Ion Source Gas2: 60 psi; Curtain Gas (CUR): 30 psi. The collision-induced ionization parameter: high.

The LC-MS data were extracted and preprocessed using MasterView (SCIEX). mainly includes the following steps: (a) metabolite feature is detected in <20% of experimental samples or detected in <50% of QC samples, it is removed from data analysis. (b) the missing values of raw data were filled up by half of the minimum value. (c) internal standard normalization method was employed in this data analysis. (d) features with RSD >30% should be removed from the subsequent analysis. The resulted three-dimensional data involving the peak number, sample name, and normalized peak area were fed to R package metaX. For principal component analysis (PCA) and orthogonal partial least square-discriminate analysis (OPLS-DA). PCA showed the distribution of origin data. In order to obtain a higher level of group separation and get a better understanding of variables responsible for classification, OPLS-DA were applied. In addition, databases including KEGG and MetaboAnalyst was utilized to search for the pathways of metabolites. There were six replicates in each group and five replicates of non-experimental standard samples were also tested. The differentially metabolites identified by both the variable importance in the projection (VIP) (> 1) and the p-value (< 0.05).

**Analysis of EPS content**

The biofilm samples were dilution in 8 mL PBS solution with heated at 80 ℃ and stirred 800 rpm/min for 1 h. Centrifuged at 10 000 rpm/min for 10 min, the supernatant filtered through 0.22 μm membrane as test sample. Two ml of the biofilm sample solution was mixed with 1 mL 6% phenol and 5 mL 98% sulfuric acid Cooling at room temperature, The absorbance was detected at *OD*_421_ nm. The polysaccharide concentrations were obtained in the light of a standard curve equation. One mL of the biofilm sample solution was mixed with 5 mL reagent A (1 g Na_2_CO_3_, 0.025 g KNaC_4_H_4_O_6_·4H_2_O and 0.2 g NaOH dissolved in 50 mL sterilized water and 0.005 g CuSO4·5H2O dissolved in 1mL of sterilized water, 50:1 mixed). After 10 min, mixed with Folin-Ciocalteu’s phenol reagent (Solarbio), react at 20-25 ℃ for 30 min, The absorbance was detected at *OD*_500_ nm. The protein concentrations were obtained in the light of a standard curve equation.

**Analysis of alginate content**

One mL of the biofilm sample was mixed with 3 mL of 10% CuSO4 solution and reaction at room temperature for 1 h, Centrifuged at 8000 rpm/min for 10 min. The precipitate was redissolved in 0.1 mL 1 mol/L NH3. H2O and added water to 1 mL. One mL of resuspension liquid was mixed with 2 mL CuSO_4_-HCl reagent (40 mL of concentrated HCl plus 1 mL of 2.5% CuSO_4_ solution plus 9 mL of water) and 1 mL of 1,3-naphthoresorcinol (100 mg of 1,3- dihydroxynaphthalene was dissolved in 25 mL of sterilized water) (Macklin) reagent. Reaction at boiling water bath for 40 min. After Cooling at room temperature, extracted with 4 mL of ethyl acetate and the organic phase was washed by 20% NaCl for 2 times. The absorbance was detected at OD421 nm. The alginate concentrations were obtained in the light of a standard curve equation.

**Analysis of rhamnolipid content**

The biofilm sample was centrifuged for 10 min at 6000 rpm/min, the supernatant was extracted with equivalent volume of ethyl acetate for twice. The upper organic phase was collected to a new tube and used liquid nitrogen to dryness, then, two hundred μL of sterile water was added to redissolve. Nine hundred of 0.19% orcinol and 53% [v/v] Concentrated sulfuric acid was added to 100 μL redissolved sample with heating at 80 ℃ for 30 min. After Cooling at room temperature, the absorbance was detected at *OD*_421_ nm. The rhamnose concentrations were obtained in the light of a standard curve equation, and the rhamnolipid concentration was calculated by multiplying the rhamnose values by a coefficient of 2.5.

**Construction of PAO1 c-di-GMP reporter strains**

First, The plasmid p*cdrA::gfpC* was purchased from addgene followed by SphⅠ/HindⅢ double digestion. The *luxCDABE* fragment was obtained by PCR using pBBR1MCS4- *luxCDABE* (Lab Store) as a template. Then, the *luxCDABE* fragment was ligated into the backbone using the Infusion one-step cloning kit (New England Biolabs). Finally, The recombinant plasmid was named p*cdrA::Lux* and was transformed into PAO1 strain. All Primers are shown in Additional file 1D.

**Motility assays**

The detected ager was consisted of peptone (1.0%), sodium chloride (1.0%), and bacto-agar (0.5%). When the culture was solidified, one μL of the biofilm sample was inoculated in the center of the agar and then incubated at 37 ℃ for 24 h. Bacterial motility was measured by diffusion diameter on the agar surface.

**Additional file 1A. The MIC of antibiotics and peptides**

| **Candidate drugs** | **MIC(μg/mL)** | **MBC(μg/mL)** | **MBEC(μg/mL)** |
| --- | --- | --- | --- |
| CRAMP | 15.625 | 31.25 | >5000 |
| LL-37 | 15.625 | - | - |
| CIP | 0.04 | 0.063 | 0.125 |
| GEN | 0.08 | 1 | 2 |

**Additional file 1B. The primer used in qRT-PCR analysis**

| **Gene name** | **Forward primer** | **Reverse primer** |
| --- | --- | --- |
| PA0763 | ATGCCGAGCTGCGTTCCACC | TCCGCCACGGTCCCTTCTCC |
| PA3385 | GCCCACTGAAACAGGCAACT | CCAGGCGAACACCGAGATTG |
| PA1432 | AAGTTGCGTGCTCAAGTGTT | CATGTAGGGGCCAGTGGTAT |
| PA1727 | GGACGGCGGACATGCGGACT | CGGCAGGCGTTTGAGATACA |
| PA2237 | CCTTCTTCAGCGAGATGCCC | CCCCACTCGGTGCTCCAGAT |
| PA3479 | ACGAGACCGTCGGCAAATAC | GCTCCAGGCAAGCCAAGTAG |
| PA3885 | TCAGCGTCCTCCGCCAGTTG | TCGTCCTCGTCGCCGAAACC |
| PA4597 | CAAACCTGCTTTCGGCGTAT | GCAGCGAGCGGTTGTTATCC |
| PA4599 | CAAGGCTGGCGACCTGCTGT | GGCGGTGGCGGTATCGAAGT |
| PA5369 | GTGCGGCAGCAAGCAGGACG | GCACCACGGAAGCCGAACCC |
| 16S | CAGCMGCCGCGGTAATWC | CCGTCAATTCMTTTRAGTTT |

**Additional file 1C. Detailed setting of Proteomic raw data processing parameters**

| **Project** | **Options** |
| --- | --- |
| Library search engine | Sequest HT |
| Database | Uniprot Database (TaxId:208964) |
| Enzyme | Trypsin |
| Miss Cleavages | 2 |
| Variable Modifications | Oxidation (M), Acetyl (Protein N-term), Deamidated (N, Q) |
| Fixed modifications | Carbamidomethyl(C) |
| Peptide Mass Tolerance | ± 10 ppm |
| Fragment Mass Tolerance | ± 0.02 Da |
| Peptide FDR | Less than 1% |
| Protein Q Value | Less than 1% |

**Additional file 1D. Primer of construction PAO1 c-di-GMP reporter strains**

| **Name** | **Orientation** | **Sequence** |
| --- | --- | --- |
| Primer 1 | **Forward** | GAGGAGAAATTAACTATGAGCATGACTAAAAAAATTTCATTCATTATTAACGGCCAGG |
|  | **Reverse** | AGTCCAAGCTCAGCTAATTAAGCTGCCATTGCACCAAAGCTAATC |
| Primer 2 | **Forward** | GTTGTAAAACGACGGCCAGTG |
|  | **Reverse** | GAATGTATGTCCTGCGTCTTGAG |

**Additional file 1E. Aamino acid sequence and structure of CRAMP and LL-37**

| **Peptide** | **Aamino acid sequence** |
| --- | --- |
| **CRAMP** | GLLRKGGEKIGEKLKKIGQKIKNFFQKLVPQPEQ  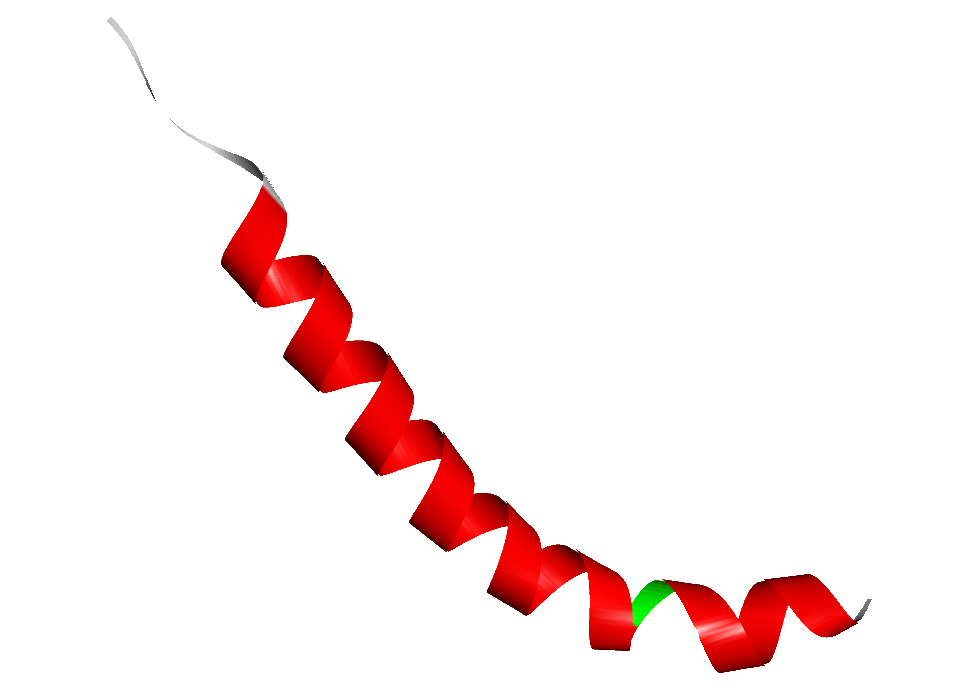 |
| **LL-37** | LLGDFFRKSKEKIGKEFKRIVQRIKDFLRNLVPRTES  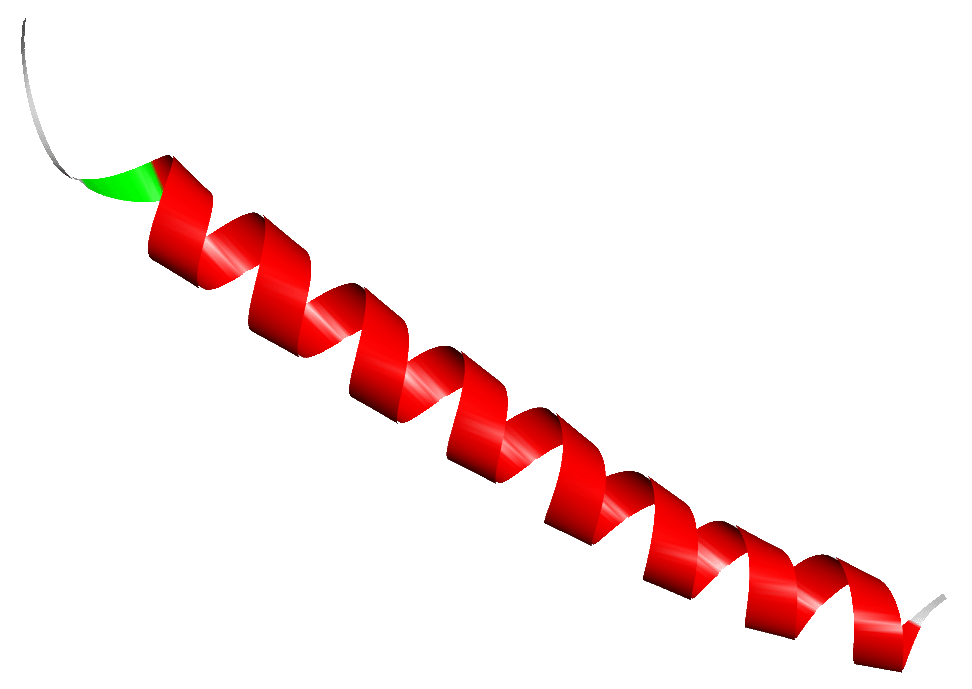 |

**
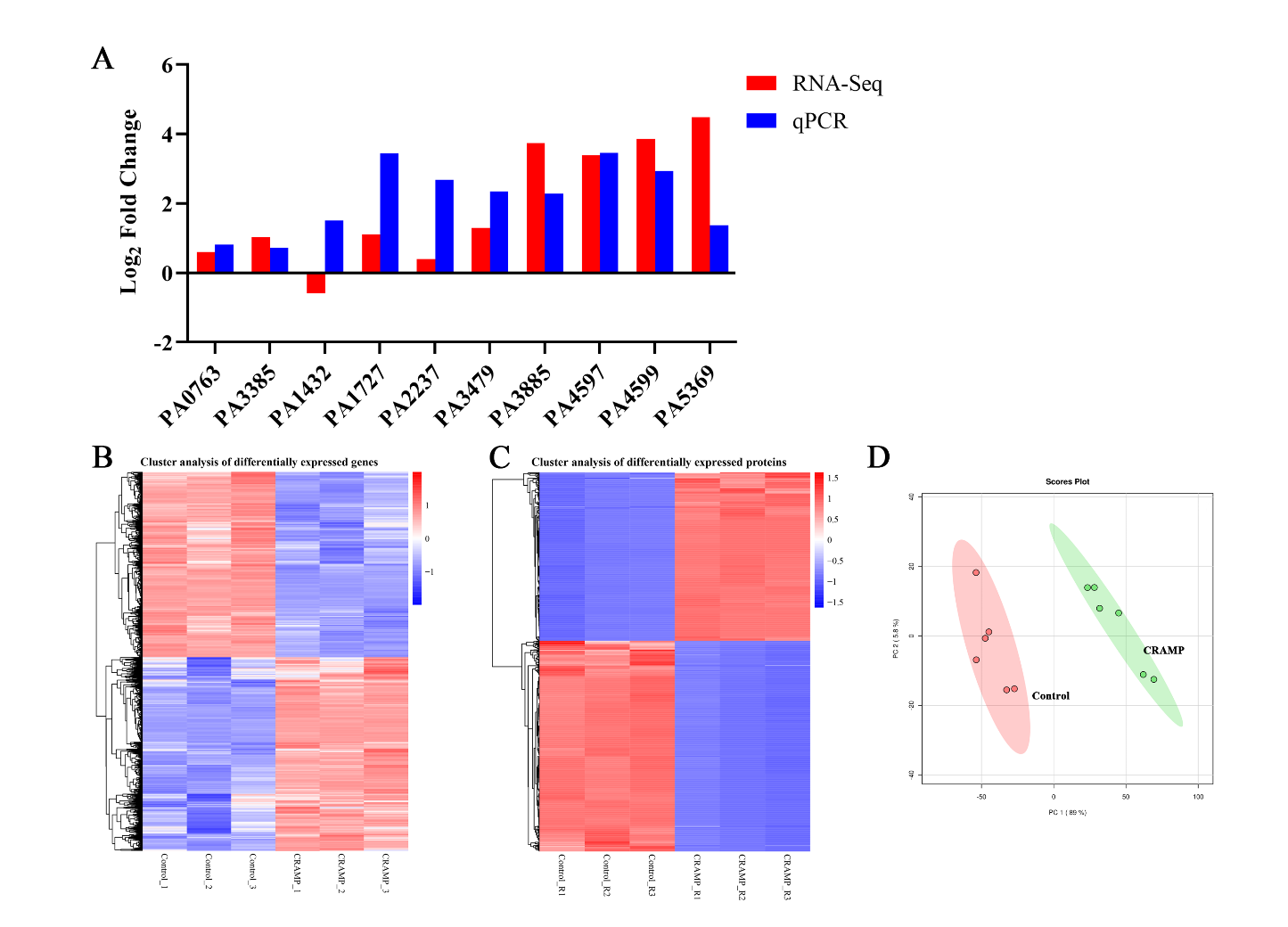
**

**Additional file 1F.** The 3-day mature PAO1 biofilm was treated with CRAMP for 1h, and the untreated PAO1 biofilm was used as the control. All experiments were performed at least in triplicate. A is the RNA sequencing data were verified by qRT-PCR of selected genes. B is clustering heat map of gene expressions based on the log10 (FPKM+1) data from transcriptomics. C is clustering heat map of protein expressions based on the the relative protein expression (logarithmically transformed data) from proteomics. D is PCA score plots of metabolic profiles based on the relative quantitative values of samples. The cluster showed six biological replicates were grouped together.

**
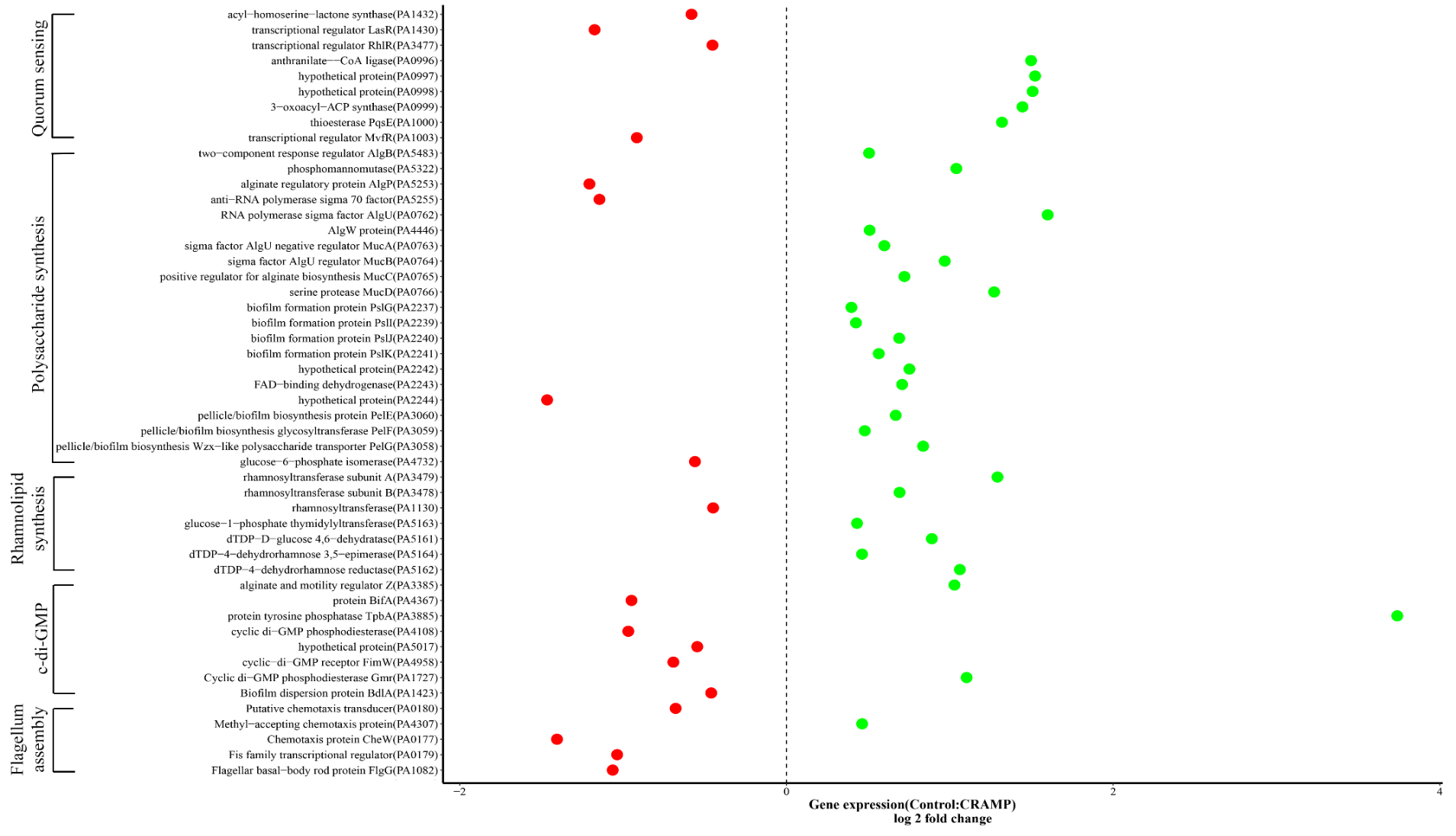
**

**Additional file 1G.** Differentially expressed genes related to biofilms in transcriptional genomics. The y-axis represents the function of each differential expressed gene and the gene's name in the NCBI database. The x-axis represents the Log_2_ Fold Change of differential expressed genes. The red dots indicate down-regulated gene expression treated with CRAMP, and the green dots indicate up-regulated gene expression treated with CRAMP. The metabolic pathway or functional classification of differential expressed genes is on the left.

**
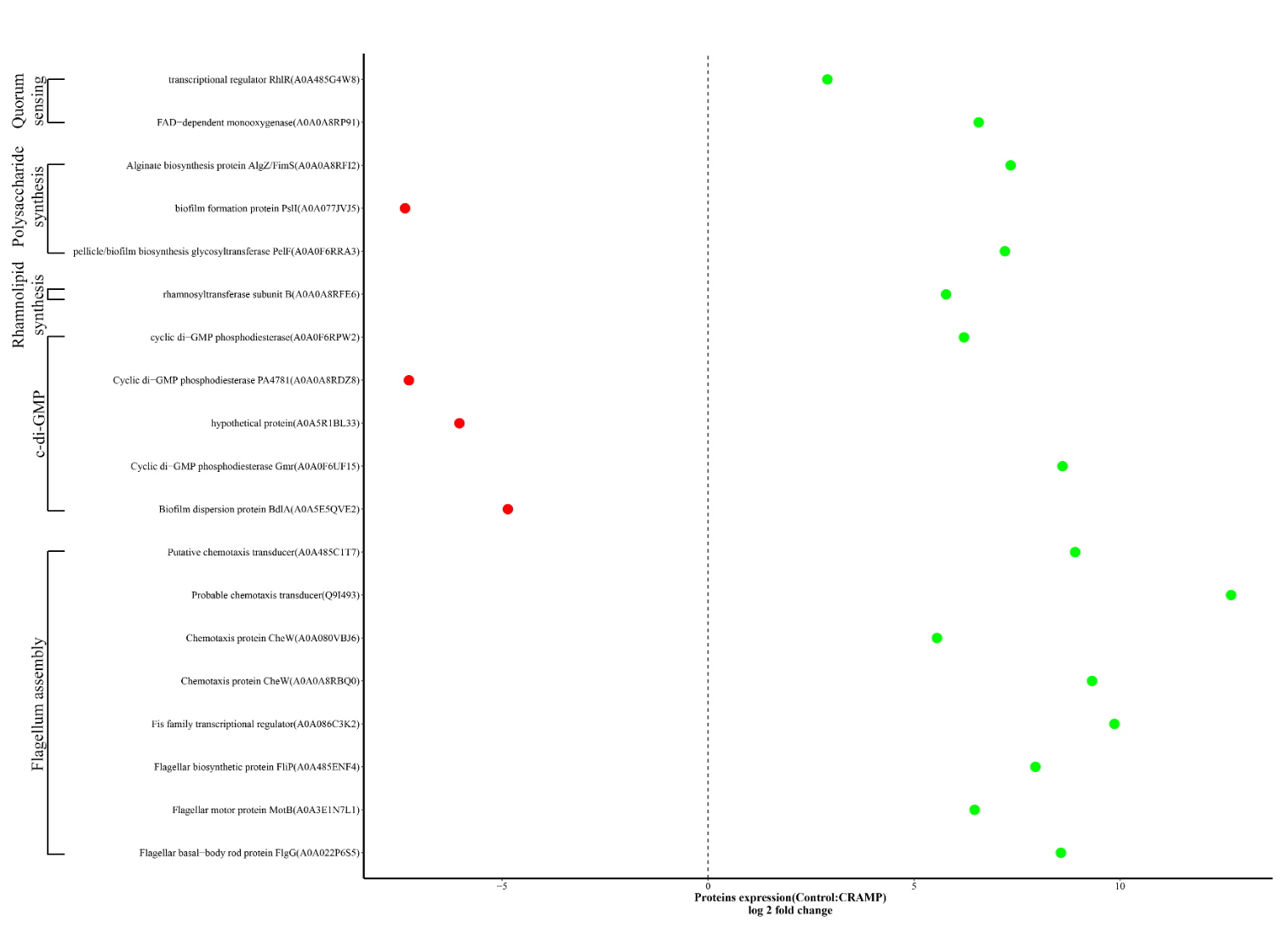
**

**Additional file 1H.** Differentially expressed proteins associated with biofilms in proteomics. The Y-axis represents the function of each differential expressed protein and the name of the protein in the Uniprot database. The X-axis represents Log2 Fold Change of differential expressed proteins. The Red dots indicate down-regulated protein expression treated with CRAMP, and the green dots indicate up-regulated protein expression treated with CRAMP. The metabolic pathways or functional classifications of relevant differential expressed proteins are on the left.
